# The soluble CD83 protein prevents bone destruction by inhibiting the formation of osteoclasts and inducing resolution of inflammation in arthritis

**DOI:** 10.3389/fimmu.2022.936995

**Published:** 2022-08-08

**Authors:** Dmytro Royzman, Darja Andreev, Lena Stich, Katrin Peckert-Maier, Andreas B. Wild, Elisabeth Zinser, Petra Mühl-Zürbes, Evan Jones, Susanne Adam, Silke Frey, Maximilian Fuchs, Meik Kunz, Tobias Bäuerle, Lisa Nagel, Georg Schett, Aline Bozec, Alexander Steinkasserer

**Affiliations:** ^1^ Department of Immune Modulation, Universitätsklinikum Erlangen, Friedrich-Alexander-Universität (FAU) Erlangen-Nürnberg, Erlangen, Germany; ^2^ Department of Internal Medicine 3, Universitätsklinikum Erlangen, Friedrich-Alexander-Universität (FAU) Erlangen-Nürnberg, Erlangen, Germany; ^3^ Fraunhofer Institute for Toxicology and Experimental Medicine (ITEM), Hannover, Germany; ^4^ Department of Medical Informatics, Friedrich-Alexander University (FAU) of Erlangen-Nürnberg, Erlangen, Germany; ^5^ Institute of Radiology, Universitätsklinikum Erlangen, Friedrich-Alexander-Universität (FAU) Erlangen-Nürnberg, Erlangen, Germany

**Keywords:** soluble CD83, arthritis, osteoclasts, metallothioneine, TLR, IDO

## Abstract

Here we show that soluble CD83 induces the resolution of inflammation in an antigen-induced arthritis (AIA) model. Joint swelling and the arthritis-related expression levels of IL-1β, IL-6, RANKL, MMP9, and OC-Stamp were strongly reduced, while Foxp3 was induced. In addition, we observed a significant inhibition of TRAP^+^ osteoclast formation, correlating with the reduced arthritic disease score. In contrast, cell-specific deletion of CD83 in human and murine precursor cells resulted in an enhanced formation of mature osteoclasts. RNA sequencing analyses, comparing sCD83- with mock treated cells, revealed a strong downregulation of osteoclastogenic factors, such as Oc-Stamp, Mmp9 and Nfatc1, Ctsk, and Trap. Concomitantly, transcripts typical for pro-resolving macrophages, *e*.*g*., Mrc1/2, Marco, Klf4, and Mertk, were upregulated. Interestingly, members of the metallothionein (MT) family, which have been associated with a reduced arthritic disease severity, were also highly induced by sCD83 in samples derived from RA patients. Finally, we elucidated the sCD83-induced signaling cascade downstream to its binding to the Toll-like receptor 4/(TLR4/MD2) receptor complex using CRISPR/Cas9-induced knockdowns of TLR4/MyD88/TRIF and MTs, revealing that sCD83 acts *via* the TRIF-signaling cascade. In conclusion, sCD83 represents a promising therapeutic approach to induce the resolution of inflammation and to prevent bone erosion in autoimmune arthritis.

## Introduction

Rheumatoid arthritis (RA) is an autoimmune disease affecting up to 1% of the world’s population, with a higher prevalence in women (1). Patients suffer from inflammation of the joints manifesting as swelling, pain, and stiffness (2). In the long term, chronic inflammation results in the degradation of cartilage and bone tissue and, if not treated properly, additional severe morbidities, such as cardiovascular disease (2). The pathogenesis of RA is multifactorial and complex, which relies on both genetic predisposition and environmental factors such as smoking and obesity (3). In the course of arthritis, RANKL is released by activated lymphocytes and promotes the formation of large multinucleated osteoclasts, which are responsible for the excessive bone resorption and systemic osteoporosis (4). The current treatment of RA primarily targets its signature cytokines TNF-α and IL-6, thereby suppressing inflammation (2, 5, 6). However, in contrast, therapeutic strategies that aim to resolve inflammation and modulate the differentiation of bone-resorbing osteoclasts during arthritis have not yet been in the focus of RA treatment.

In this respect, the soluble CD83 (sCD83) molecule represents a very interesting option since we and others have shown that sCD83 modulates autoimmune-mediated disorders and induces the resolution of inflammation, a prerequisite to guarantee a long-term cure for patients suffering from RA (7–10). The sCD83 molecule is composed of the extracellular domain of the membrane-bound mCD83 isoform, which is expressed on activated immune cells, including DCs, B and T cells as well as regulatory T cells, and is cleaved from the cell surface by a yet unknown mechanism (11–14). Interestingly, this immune modulatory molecule has been associated with arthritis since Hock et al. reported significantly increased levels of sCD83 in the synovial fluids of RA patients (15). However, its biological function and, in particular, its therapeutic potential remained unclear until our recent work, which unveil the inhibitory effects of sCD83 on arthritis (8).

Mechanistically, sCD83 mediates its protective effects *via* activating the enzymatic activity of indoleamin-2,3-dioxygenase (IDO) (16). The increased conversion of the amino acid tryptophan into kynurenine results in (i) impaired effector T cell differentiation and (ii) reduced survival due to tryptophan starvation (17). Concomitantly, kynurenine is a potent inducer of regulatory T cells, which are crucial for the resolution of inflammation and inhibiting osteoclast formation (17, 18). In the current project, we focused on the effect of sCD83 on inducing the resolution of inflammation and inhibiting osteoclast formation in established arthritis. We also explored by which signaling sCD83 promoted pro-resolving effects in arthritis and translated the findings to human by testing sCD83-inhibited osteoclastogenesis in patient-derived samples.

## Materials and methods

### Mice

Female C57BL/6 mice (6–8 weeks old) were purchased from Charles River Laboratories (Sulzfeld). For an “osteoclast-specific” deletion of CD83, we crossed CD83^fl/fl^ mice, which were generated as previously described (12), with a Cx3cr1-Cre line [official name: STOCK Tg(Cx3cr1-cre)MW126Gsat/Mmucd], which was kindly provided by Prof. Krönke (Department of Medicine 3, University Hospital Erlangen, Erlangen, Germany). The mice were maintained under pathogen-free conditions according to the institutional and national guidelines for the care and use of laboratory animals. All studies were approved by the animal ethical committee of the government of Unterfranken, Würzburg.

### Antigen-induced arthritis model

C57BL/6 mice were pre-immunized at day −21 and −14 by s.c. injection of 100 μl complete Freund’s adjuvant emulsion (Sigma-Aldrich) enriched with 10 μg/ml heat-killed *Mycobacterium tuberculosis* strain H37RA (Difco) and methylated bovine serum albumin (mBSA; Sigma-Aldrich) in a final concentration of 1 mg/ml. Along with the immunization, 200 ng *Bordetella pertussis* toxin (Quadratech) was administered intraperitoneally (i.p.) in 100 μl phosphate-buffered saline (PBS; Lonza). The effector phase was induced on day 0 by the intra-articular (i.a.) injection of 100 μg mBSA into the right knee of the anesthetized mice. The left knee was injected with PBS as an internal control. Flare-up reaction was induced by a second i.a. mBSA injection on day 7, analogous to the first i.a. injection, and 100 µg sCD83 or PBS (as non-treated controls) was administered systemically on days 8, 9, and 10 by i.p. injection. Knee joint swelling was assessed from the time of induction (day 0) up to day 17 using a JD 50 TOP caliper (Käfer Messuhrenfabrik) in a randomized and blinded manner. The maximum medial-to-lateral diameter was defined at the widest point of each knee joint. Knee joint swelling was calculated as the absolute difference to the knee joint diameter determined at baseline before arthritis induction and expressed as the percentage of knee joint swelling. The mice were euthanized by cervical dislocation on day 17.

### Histological analyses and arthritic score

Bone tissue was fixed overnight in 10% formalin, decalcified for 2 h in Osteomoll (Merck), and embedded in paraffin. Paraffin sections (5 μm) were stained with Safranin O for the morphological evaluation of inflammatory cell influx, synovitis, cartilage degradation (proteoglycane content), and bone resorption. A score of 5 represents the highest score, while 0 represents a healthy score, without any histological abnormalities. Scoring was performed in a blinded fashion.

### Micro-CT analysis

The hind limbs were isolated, fixed overnight in 10% formalin solution at 4°C (Sigma-Aldrich), and stored in 70% ethanol until μCT measurements. Imaging was performed using a dedicated preclinical scanner (Inveon, Siemens Healthineers) at a tube voltage of 80 kV and a tube current of 500 μA. Images were acquired with an isotropic resolution of 50.67 μm for measuring of mean density values within the femoral epiphyses and with 8.98 μm for high-resolution 3D surface reconstructions. For image analyses, 3D multiplanar reconstructions were generated using the freeware DICOM viewer Osirix (21), with a total of six regions of interest (ROI) placed in the femoral epiphysis of each animal (two in paraaxial, two in paracoronal, and two in parasagittal orientation). The target size of each ROI was 0.5 mm^2^. The mean density measures (Hounsfield units) of the six ROI were assessed and averaged for each animal. For 3D surface reconstructions, the Volume Rendering Technique included in the syngo.via package (version V20A, Siemens Healthineers) was used.

### Osteoclastogenesis

#### Mouse-derived cells

Total bone marrow cells were isolated from C57BL/6 mice (7 weeks) by flushing the femur and tibia. Blood cells were lysed using 0.8% ammonium chloride for 5 min at 37°C. The cells were plated overnight in OC medium (αMEM + GlutaMAX-I (Gibco) + 10% fetal calf serum (FCS)/1% PS) supplemented with 5 ng/ml M-CSF (Peprotech). Non-adherent bone marrow-derived monocytes (BMMs) were collected, washed, and further cultured in OC medium supplemented with 20 ng/ml M-CSF and 20 ng/ml RANKL (Peprotech) in 96-well plates at a density of 1 × 10^6^ cells/ml. The medium was changed on day 2. These cells were incubated daily with 25 μg/ml sCD83 from day 1 onwards. For experiments on the therapeutic potential of sCD83, 25 µg/ml sCD83 was only added on day 2 to the culture medium. At day 3, fully differentiated osteoclasts were washed with PBS and fixed with fixation buffer (25 ml citrate buffer + 65 ml acetone + 8 ml 37% PFA). Osteoclast differentiation into mature multinucleated TRAP^+^ cells (≥15 nuclei) was evaluated by TRAP staining using the leukocyte acid phosphatase kit 386A (Sigma-Aldrich) according to the manufacturer’s instructions. For RNA analyses, the cells were lysed in QIAGEN’s RLT PLUS buffer containing 1% β-mercaptoethanol.

#### Human-derived cells

Peripheral blood mononuclear cells (PBMCs) were either isolated from leukoreduction system chambers (LRSCs) from healthy donors or from blood samples of RA patients according to a published protocol (19). In total, 3 × 10^5^ cells were plated in 100 µl adherence medium [αMEM + GlutaMAX-I (Gibco) + 1% FCS/1% PS] per well of a flat-bottomed 96-well plate for 90 min (5.5% CO_2_ and 37°C). Non-adherent cells were removed by flushing, and residual cells were cultured in 200 µl of OC medium [αMEM + GlutaMAX-I (Gibco) + 10% FCS/1% PS] supplemented with 30 ng/ml hu M-CSF (Peprotech), 10 ng/ml hu RANKL (Peprotech), and 1 ng/ml hu TGFβ (BioLegend) at 5.5% CO_2_ and 37°C. The OC medium was replaced on days 3 and 6. Moreover, 25 µg sCD83 or the corresponding volume of PBS was added on days 0, 3, 4, 5, and 6. On day 7, fully differentiated osteoclasts were processed analogously to the murine protocol for TRAP staining and RNA extraction.

### Osteogenic induction of MSCs and staining of calcium nodules

Isolation of C57BL/6 mesenchymal stem cells (MSCs) was performed as described elsewhere (20), in accordance with the MSC minimal criteria (21). Cells were maintained in a growth medium [α-MEM with 100 U/ml penicillin and 100 µg/ml streptomycin (all Gibco) and 10% FCS [(Biochrome)]. For osteogenic induction, MSCs were plated in 35-mm cell culture dishes at a density of 3.800 cells/cm² and a volume of 2.5 ml. When the cells were 100% confluent, the medium was changed to mineralization medium [MSC growth medium, with 568 µM L-ascorbic acid 2-phosphate sesquimagnesium salt hydrate (Sigma-Aldrich) and 5 mM β-glycerol phosphate disodium salt pentahydrate (Calbiochem)]. The mineralization medium was supplemented with either sCD83 (25 and 10 µg/ml, as indicated), PBS (vehicle control), or no additional solvent (w/o) and changed every two days. Staining for calcium nodules with Alizarin Red was performed on day 9. For that, the cell culture medium was discarded, and the cells were fixed with 95% ethanol. The wells were then washed with deionized (DI) H_2_O, stained with 2% Alizarin Red solution (pH 4.2, Sigma-Aldrich) for 2 min, and repeatedly washed with DI H_2_O afterwards. Photographs of the wells were taken and analyzed with image analysis software (Adobe Photoshop CS6 version 13.0.1). The ratio of mineralized surface area in relation to the whole surface area was quantified as a measurement of osteoblastic mineralization activity.

### CRISPR/Cas9-mediated gene knockout

The experimental procedure of CRISPR/Cas9-mediated knockout in human- and mouse-derived primary cells was adapted from Hiatt et al. (22) and Freund et al. (23). Briefly, PBMCs (human) or bone marrow cells were isolated and purified as described previously, and 1 × 10^6^ MACS-sorted CD14^+^/CD16^-^ monocytes (human; Classical Monocyte Isolation Kit; Miletnyi Biotec) or 1 × 10^6^ non-adherent BMM cells (mouse) were used per nucleofection reaction. The cells were spun down at 300*g* for 10 min. The supernatant was completely removed, and the cells were resuspended in 20 μl/reaction of room-temperature primary nucleofection buffer P2 (Lonza) for human-derived samples and primary nucleofection buffer P3 (Lonza) for murine cells. The cell suspension was gently premixed with 5 μl reaction mixture containing 2 µl sgRNA (IDT) + 2 µl Alt-R^®^ S.p. Cas9 Nuclease V3 (IDT) + 1 µl PBS per 1 × 10^6^ cells and then pipetted into a 16-well-format nucleofection stripe for the Lonza 4D X unit or Shuttle unit (Lonza). Analogously, the control cells were nucleofected with the negative control sgRNA from the Alt-R^®^ CRISPR-Cas9 Control Kit (mouse or human; IDT), respectively. The cassettes were nucleofected with code DK-100 for human-derived samples and CM-137 for murine cells. Immediately after nucleofection, the wells were filled up to 60 μl with pre-warmed αMEM for 5 min at 37°C. Subsequently, 1 × 10^6^ cells were split into two wells of a 96-well flat-bottomed culture plate containing 170 µl of pre-warmed αMEM and kept for an additional 2 h at 37°C. Afterwards, αMEM was carefully exchanged for 200 µl of OC differentiation medium. The cells were further handled according to the corresponding osteclastogenesis protocol.

### Evaluation of genome targeting efficiency using flow cytometry or T7 endonuclease

In order to determine the CRISPR/Cas9-mediated CD83-specific knockout efficiency on human-derived CD14^+^/CD16^-^ cells, 1 × 10^6^ of nucleofected cells were differentiated into dendritic cells, which highly express the CD83 molecule on their surface. The reason for the use of mature DCs to analyze the KO efficacy is that mature osteoclasts lose their CD83 surface expression and are thus not suitable. Briefly, nucleofected cells were allowed to adhere to plastic tissue culture plates (Falcon, Fischer Scientific, Germany) for 2 h at 37°C. Afterwards, the supernatant was removed carefully, and the wells were washed three times with pre-warmed RPMI 1640 (Lonza). The mononuclear cells were inoculated for 5 days in a medium consisting of RPMI 1640 (Lonza, Basel, Switzerland), supplemented with 1% (v/v) heat-inactivated human serum type AB (Sigma-Aldrich), 1% (v/v) penicillin streptomycin L-glutamine (Sigma-Aldrich), and 10 mM HEPES (Lonza) in the presence of 800 IU/ml (day 0) or 400 IU/ml (day 3) recombinant human GM-CSF and 250 IU/ml (days 0 and 3) recombinant human IL 4 (both from Miltenyi Biotec, Bergisch Gladbach, Germany). On day 4, the cells were matured with 100 ng/ml lipopolysaccharide (LPS; Sigma Aldrich) for 16 h. To assess the efficiency of CRISPR/Cas9-mediated knockdown on protein level, LPS-matured human mDCs were analyzed by FACS on day 5, regarding their CD83 cell surface expression, using a specific monoclonal antibody (CD83-PE, clone HB15e BioLegend) diluted in Dulbecco’s phosphate-buffered saline. For dead cell discrimination, 7-AAD viability staining solution (ThermoFisherScientific) was added to the mixture, and cells were incubated for 30 min at 4°C.

Regarding the CRISPR/Cas9-mediated knockdown of TLR4, MyD88, TRIF, and MT-1/MT-2, which are intracellular molecules or are internalized during the osteoclastogenesis process, targeting efficiency was determined using the T7 Endonuclease I assay according to the instructions of the manufacturer (New England Biolabs). Briefly, the DNA region around the CRISPR/Cas9-mediated double-strand break was PCR-amplified using specific primer pairs (**Table 1**), and the PCR product was further purified using Qiagen’s PCR-Purification Kit. In total, 200 ng DNA was initially denatured and allowed to form heteroduplexes during the cool-down. T7-Endonuclease I digestion was performed at 37°C for 15 min, and the samples were loaded onto 2% agarose gel for separation. Knockdown efficiency was assessed according to the band intensity of digested DNA heteroduplexes relatively to the naïve band intensity using ImageJ software.

**Table 1:** Targeting sequences used for CRISPR/Cas9-mediated gene knockouts as predesigned by IDT’s guide RNA tool (first two columns) along with specific primers which were used in order to determine the knockout efficiency (last three columns, 5′ → 3′).

Gene	Target sequence		Forward	Reverse
CD83	GCTGCAACTCGGGGACATAC	huCD83	ATCCAGCAAGATTCACACACAGC	CGGATTTCCACTTTCAGCCACA
TLR4	TCTGACGAACCTAGTACATG	muTLR4	CCAGCCAGGTTTTGAAGGCAA	GTATCTCTTTTGCCCATAGGTGTGA
TRIF/TICAM1	CAAGCTATGTAACACACCGC	muTRIF/TICAM1	CTCGTCCCATTGACACACCA	AGGGGAGACCTGGAGTTTGT
MyD88	TCCCACGTTAAGCGCGACCA	muMyD88	AACTCCACAGGCGAGCGTA	TGGAAGGGGTCCTCACTTGT
MT-1	TCGTCCAACGACTATAAAGA	muMT-1	CTCTCTGGAGGGAGGGTACC	AGCCTCTACAACTCGGGGAT
MT-2	GCGTGATGGAGAGAAGCACG	muMT-2	TGTGCTGGCCATATCCCTTG	GTCGGAAGCCTCTTTGCAGA

### qPCR analyses

Total RNA was isolated from the synovial tissue of AIA mice or 2 × 10^6^ cells from osteoclast cultures using RNeasy Plus Mini Kit (Qiagen) according to manufacturer’s protocol. In terms of CRISPR/Cas9-nucleofected samples, gDNA eliminator columns were not discarded, but DNA was eluted from the column membrane for the subsequent evaluation of knockout efficiency. An additional DNase digestion step was performed in order to remove residual DNA. cDNA synthesis was performed according to First Strand cDNA Synthesis Kit manual (Thermo Fisher Scientific). The PCR mixture contained 5 μl SsoAdvanced Universal SYBR Green Supermix (Biorad), 0.5 μl of 5′ and 3′ primer (forward and reverse, each at 1 μM), and 2.5 μl RNA reverse transcription product corresponding to 12.5 ng of cDNA. The reaction was adjusted to 10 μl with *aqua ad iniectabilia* (Braun). After an initial denaturation step at 95°C for 3 min, 45 cycles of denaturation (94°C for 15 s), annealing (61°C for 15 s), and extension (72°C for 15 s) were performed using Bio-Rad CFX96 Touch Real-Time PCR Detection System (Biorad). Gene expression was calculated relatively to the housekeeping gene Rpl4 (Ribosomal Protein L4) for the murine or Rpl13A (Ribosomal Protein 13A) for the human osteoclast experiments. The primer sequences are listed in **Tables 1**, **2**.

**Table 2:** Sequences of primer pairs (in 5′ to 3′ direction) used within the current work.

	Forward	Reverse
**(A) Human**		
TRAP	TGAGGACGTATTCTCTGACCG	CACATTGGTCTGTGGGATCTTG
Oscar	AGATCGCTCCCCTTCTCTTC	TAGCAGCAGCGGTAACTTCC
MMP9	CCTGGAGACCTGAGAACCAA	ATTTCGACTCTCCACGCATC
Nfatc1	GTCCTGTCTGGCCACAAC	GGTCAGTTTTCGCTTCCATC
RANK	TCCTCCACGGACAAATGCAG	CAAACCGCATCGGATTTCTCT
Ctsk	AGAAGACCCACAGGAAGCAA	GCCTCAAGGTTATGGATGGA
IFNβ	GCGACACTGTTCGTGTTGTC	GGCAGTATTCAAGCCTCCCA
**(B) Murine**		
RANK	TTGTGGCAGGGGACTTTAAC	ATTGTCATCCTGCCCTCAAC
MMP9	GCTGACTACGATAAGGACGGCA	TAGTGGTGCAGGCAGAGTAGGA
OC-Stamp	TTGCTCCTGTCCTACAGTGC	GCCCTCAGTAACACAGCTCA
DC-Stamp	TGGAAGTTCACTTGAAACTACGTG	CTCGGTTTCCCGTCAGCCTCTCTC
Nfatc1	GGTGCCTTTTGCGAGCAGTATC	CGTATGGACCAGAATGTGACGG
Beta-actin	TGTCCACCTTCCAGCAGATGT	AGCTCAGTAACAGTCCGCCTAGA
IL-1β	TGCCACCTTTTGACAGTGATG	ATGTGCTGCTGCGAGATTTG
IL-6	ACAAAGCCAGAGTCCTTCAGAG	GAGCATTGGAAATTGGGGTAGG
RANKL	CAGCCATTTGCACACCTCAC	CCCGATGTTTCATGATGCCG
Foxp3	CCCAGGAAAGACAGCAACCTT	CCTTGCCTTTCTCATCCAGGA

**(A)** Mouse-specific primers. **(B)** Human-specific primers.

### RNA sequencing experiments

#### RNA sequencing was performed by Novogene

Raw paired-end reads were aligned to the reference genome (GRCh38) using Rsubread (v. 2.6.4) (24) within R programming language (v. 4.1.1). Quantification was performed using feature Counts function from RSubread. Differential expression analysis was performed following the previously published pipeline (25) using DESeq2 (v 1.32.0) (26). g:Profiler web-tool (27) was used for the functional analysis of differentially expressed genes.

### Statistical analyses

All data are expressed as mean ± SEM. Normal distribution was verified by Shapiro–Wilk test, and statistical significance was calculated using Student’s *t*-test for single comparison or Mann–Whitney *U*-test for nonparametric distribution. The grouped data were analyzed using one- or two-way ANOVA followed by Sidak’s multiple-comparison test. All calculations were performed using GraphPad Prism 8 (GraphPad). *P*-values <0.05 were considered significant. Asterisks mark the statistically significant differences (*p < 0.05, **p < 0.01, ***p < 0.001, and ****p < 0.0001). The absence of asterisks indicates that there is no statistical significance.

## Results

### Therapeutic administration of sCD83 ameliorates arthritic symptoms and accelerates the resolution of inflammation

**Figure 1:**
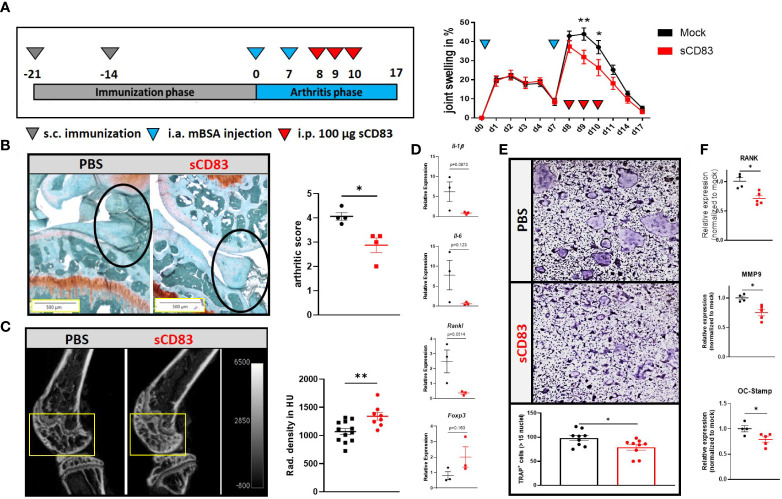
Therapeutic treatment with sCD83 prevents excessive bone destruction *in vivo* as well as *in vitro*. **(A)** Schematic overview of the applied antigen-induced arthritis (AIA) model (**A**; left-hand side). Percent increase of knee swelling (normalized to baseline) after the local intra-articular injection of mBSA and sCD83 treatment (*n* = 9) or a vehicle control (*n* = 10) (**A**; right-hand side). **(B)** Histologic arthritis score (mean value of inflammation, synovitis, cartilage destruction, and bone resorption) was assessed from Safranin O-stained 5-µm joint slides derived from sCD83- or PBS- treated AIA mice on day 17 with *n* = 4 each, wherein 5 represents the highest score and 0 represents healthy animals. **(C)** Representative μ-computed tomography images of knee joints (left-hand side) and quantification of bone mass of femoral epiphysis [right-hand side with sCD83 (*n* = 8) and phosphate-buffered saline, PBS (*n* = 10)]. **(D)** RT-PCR analyses of synovial RNA expression levels of arthritis-related gene transcripts. *Rpl4* was used as a housekeeping gene (*n* = 3). € Upper part: representative images used for the quantification of large multinucleated TRAP^+^ cells with more than 15 nuclei. Lower part: quantification of PBS- and sCD83-treated samples with *n* = 9. **(F)** RT-PCR analyses regarding the expression of osteoclast-related gene transcripts from a representative experiment [sCD83 (*n* = 5) and PBS (*n* = 4)]. **(A)** Two-way ANOVA, **(D)** Student’s *t*-test, and **(B, C, E, F)** Mann–Whitney test.

Based on our previous work, where sCD83 revealed a striking anti-arthritic potential in the AIA model when applied prophylactically during the immunization phase, we now assessed the therapeutic potential of sCD83 in inducing the resolution of arthritis (8). AIA was induced in female C57BL/6 mice, and the mice were rechallenged with a flare-up reaction by a second i.a. mBSA injection into the knee joints (28). Only during this late arthritic phase, sCD83 was applied therapeutically (100 µg/day/i.p.) on days 8, 9, and 10, which correlates with the peak of inflammation (**Figure 1A**). As depicted in **Figure 1A** (right-hand side), a significantly reduced joint swelling was observed on days 9 and 10, thus already one day after the first sCD83 application on day 8. Concomitantly, histological analyses of the joints of sCD83-treated animals revealed a significantly ameliorated arthritic score as visualized by Safranin O staining (**Figure 1B**). Although both groups have already faced a severe loss of cartilaginous and bone tissue in the course of the first arthritic phase, a prominent difference was observed regarding the influx of inflammatory cells (see **Figure 1B**, black circles). Since sCD83 was only applied during the late arthritic phase and not during the immunization phase, cartilage destruction and bone resorption took place already (**Figure 1B**). However, the µCT imaging analyses of arthritic joints showed a highly significant increased bone density in samples derived from sCD83-treated animals (**Figure 1C**).

Next, we assessed if and how sCD83 treatment modulates the local cytokine milieu using RT-PCR analyses of the arthritic synovial tissue. As shown in **Figure 1D**, pro-inflammatory cytokines such as *Il-1β* and *Il-6* as well as the pro-osteoclastogenic factors *Rankl* were down-modulated on day 17. In contrast, *Foxp3* expression was slightly increased in sCD83-treated mice, possibly indicating a higher percentage of Tregs present within the synovial tissue of sCD83-treated animals (**Figure 1D**). Based on these results and the observed reduced bone loss in sCD83-treated animals (**Figures 1C, D**), we assessed the capacity of sCD83 to modulate mature osteoclasts, which mimics the situation present in RA patients during a flare-up reaction. For this purpose, osteclastogenesis was induced in bone marrow-derived cells (BMDCs), and sCD83 (25 µg/ml) was applied on already maturing osteoclasts for 24 h. As shown in **Figure 1E**, one dose of sCD83 efficiently hampered the formation of large multinucleated osteoclasts beyond the mononuclear or pre-osteoclast cell status. In addition, the presence of sCD83 in the osteoclast culture medium resulted in a significant reduction of osteoclast-related transcripts such as *Rank*, *Mmp9*, and *Oc-Stamp* (**Figure 1F**).

### Depletion of CD83 promotes the formation of large multinucleated and active osteoclasts

**Figure 2:**
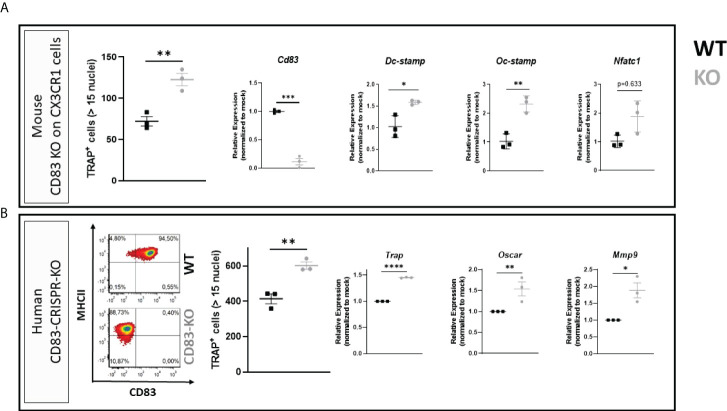
Membrane-bound CD83 expression protects from excessive osteoclastogenesis in mice and men. **(A)** Osteoclasts were generated from conditional CD83 knockout mice, where CD83 was specifically depleted on CX_3_CR_1_-expressing cells. The number of large multinucleated TRAP^+^ cells with more than 15 nuclei was assessed (**A**; far left-hand side) with phosphate-buffered saline (PBS) at *n* = 3 and sCD83 at *n* = 3. RT-PCR analyses regarding the expression of CD83 and osteoclast-related genes normalized to WT value (**A**; right-hand side; sCD83 at *n* = 3 and PBS at *n* = 3). **(B)** CD83 was knocked down in peripheral blood mononuclear cell (PBMC)-derived cells *via* CRISPR/Cas9 method, and cells were then used for osteoclastogenesis experiments. Knockdown efficacy was determined by flow cytometry of surface CD83 expression on mature dendritic cells derived from the same targeted PBMC stock, which were differentiated into osteoclasts (**B**; left-hand side). A representative image from out of three independent experiments is shown on the left-hand side. The number of large multinucleated TRAP^+^ cells with more than 15 nuclei was assessed (**B**; middle) with PBS at *n* = 3 and sCD83 at *n* = 3. The RNA levels of osteoclast-related gene transcripts and CD83 were determined by RT-PCR and normalized to the corresponding mock-nucleofected sample of the same donor in order to account for donor-specific variations (**B**; right-hand side with *n* = 3). Student’s *t*-test analyses were performed.

Based on these data, we addressed the biological role of the membrane-bound CD83 (mCD83) isoform during osteoclastogenesis. For this purpose, BMDCs were isolated from mice, where CD83 was specifically depleted on CX_3_CR_1_-expressing cells (*i*.*e*., monocytes, macrophages, and osteoclasts). Surprisingly, the number of large TRAP^+^ osteoclasts was significantly increased when CD83 was absent (**Figure 2A**; see TRAP^+^ cells). Moreover, transcriptome analyses revealed a significant induction of osteoclast fusion-related transcripts, such as *Dc-Stamp* and *Oc-Stamp* (**Figure 2A**). Next, we aimed to translate these murine observations into the human system using the CRISPR/Cas9 technology to delete CD83 expression in human monocytes, which were then differentiated into osteoclasts. CD14^+^/CD16^-^ cells were MACS-isolated from human PBMCs and nucleofected with CD83-specific or control sgRNA along with the Cas9 protein. One part of the nucleofected cells was differentiated and stimulated towards CD83^+^ mature dendritic cells in order to determine the knockout efficacy by flow cytometry. As depicted in **Figure 2B**, CD83 surface expression was diminished from 94.5% on control cells down to 0.4% in targeted cells. Residual cells were used for osteoclastogenesis experiments, and CD83 deletion revealed a significantly enhanced osteoclastogenic potential (see TRAP^+^ cells in **Figure 2B**), like murine CD83-knockout cells (**Figure 2A**). The depletion of CD83 did not only increase the number of large TRAP^+^ multinucleated osteoclasts but also significantly upregulated the expression of osteoclast-related genes such as *Trap*, *Oscar*, and *Mmp9* (**Figure 2B**; right-hand side). Taken together, these data demonstrate that CD83 is an important factor for osteoclast differentiation, fusion, and their activity.

### Effects of sCD83 on human osteoclast gene expression

**Figure 3:**
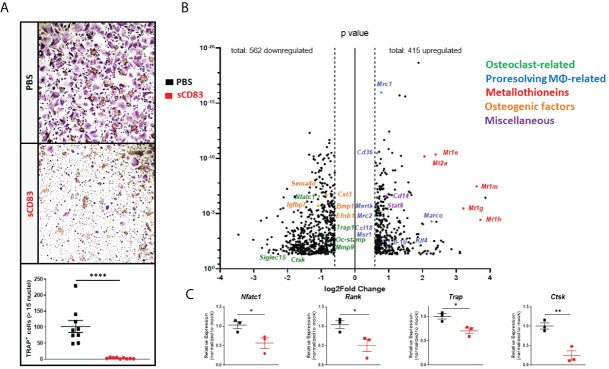
sCD83 treatment blocks osteoclast formation from human-derived peripheral blood mononuclear cells. **(A)** Representative image (**A**; upper side) used for the quantification of large multinucleated TRAP^+^ cells with more than 15 nuclei (**A**; lower side) with *n* = 9. **(B)** Volcano plot of RNA sequencing analyses of sCD83- *vs*. PBS-treated osteoclasts from healthy donors on day 7, derived from three independent donors per condition. The dots depicted on the right-hand side of the log2Fold change 0 value represent significantly upregulated gene transcripts, while the dots on the left-hand side represent significantly downregulated transcripts with log_2_FC ≥0.6, respectively. Specific dot colors classify the transcripts into characteristic groups: green = osteoclast-related, blue = transcripts associated with alternatively activated M2-like MΦ, red = metallothioneins, orange = transcripts which are associated with osteoblast formation, and purple = miscellaneous transcripts, with a prominent effect on monocytes, MΦ, or osteoclasts. **(C)** Verification of the RNA sequencing data by RT-PCR analyses regarding the expression of osteoclast-related transcripts from one representative experiment (*n* = 3). **(A)** Mann–Whitney test and **(C)** Student’s *t*-test. Asterisks mark the statistically significant differences (*p < 0.05, **p < 0.01, ***p < 0.001, and ****p < 0.0001).

To further analyze the influence of sCD83 regarding osteoclast differentiation on a transcriptional level, PBMCs were isolated from LRSCs and used for osteoclastogenesis experiments in the presence of sCD83 (25 µg/ml) or PBS. On day 7, the number of large multinucleated osteoclasts was determined by TRAP staining, and it revealed a significant reduction of large multinucleated osteoclasts in the presence of sCD83 (**Figure 3A**).

Concomitantly, RNA was isolated from sCD83- and mock-treated osteoclasts on day 7 for RNA sequencing. The evaluation of these sequencing analyses showed a downregulation of 562 mRNA targets along with an upregulation of 415 transcripts by sCD83 treatment when compared to PBS-treated controls (**Figure 3B**). We have identified five prominent clusters which play a pivotal role in the context of osteoclasts and bone homeostasis, *i*.*e*., osteoclasts-related- (green), M2-like MΦ-related- (blue), metallothioneins- (red), osteogenic factors- (orange), and miscellaneous- (purple) transcripts, which are associated with inflammatory events or the osteoclastogenesis process. Interestingly, osteoclast-related transcripts *Nfact1*, *Trap*, *Oc-Stamp*, *Mmp9*, and *Ctsk* along with *Siglec15* were all found to be significantly downregulated in sCD83-treated samples (**Figure 3B**). These sequencing data were subsequently verified by RT-PCR analyses and confirmed, *e*.*g*., the downregulation of *Nfatc1*, *Rank*, *Trap*, and *Ctsk* (**Figure 3C**) (8, 29).

Unexpectedly, the sequencing analyses also revealed a prominent upregulation of metallothioneins (*i*.*e*., 1E/1M/1G/1H and 2A) following sCD83 treatment. Interestingly, metallothioneins are key players in inflammation and osteoclastogenesis *via* their binding of free zinc ions (Zn^2+^) and the stabilization of the anti-inflammatory TRIF-signaling cascade (30, 31). Furthermore, a strong upregulation of alternatively activated M2-like MΦ markers, such as *Mrc1*, *Cd36*, *Mertk*, *Mrc2*, *Msr1*, *Klf4*, *Il-10*, and *Marco*, was induced in the presence of sCD83, representing the induction of a shift towards alternatively activated M2-like MΦ (32–34). In addition, we have identified “miscellaneous” transcripts which negatively affect the osteoclastogenesis process. This includes, *e*.*g*., *Cd14*, indicating a sCD83-mediated suppression of the differentiation process which results in higher numbers of non-differentiated precursor monocytes, while the signal transducer and activator of transcription 6 (*Stat6)* reportedly blocks the RANK-induced osteoclast differentiation process (**Figure 3B**) (35, 36). Furthermore, we identified a significant reduction of osteogenic factors, such as *Sema4d*, *Igfbp7*, *Cst3*, *Bmp1*, and *Efnb1*, that are released by osteoclasts in the course of the physiological bone renewal process, indicating that sCD83 treatment does not go along with a risk of constitutive osteoblast induction and excessive bone formation (**Figure 3B**) (37–39).

### Effects of sCD83 on osteoblast differentiation

Finally, in this respect, we also investigated whether or not sCD83 affects osteoblast differentiation and/or activity using a protocol whereby osteoblasts were differentiated from mesenchymal stem cells. However, as shown in **Supplementary Figure S1**, the presence of sCD83 neither affected osteoblast formation nor the mineralization activity of osteoblasts. In summary, these data clearly indicate that, in cells generated from healthy donors, sCD83 represents a pivotal checkpoint molecule for the generation/differentiation of human osteoclasts.

### Effects of sCD83 on the gene expression of osteoclast from rheumatoid arthritis patients

**Figure 4:**
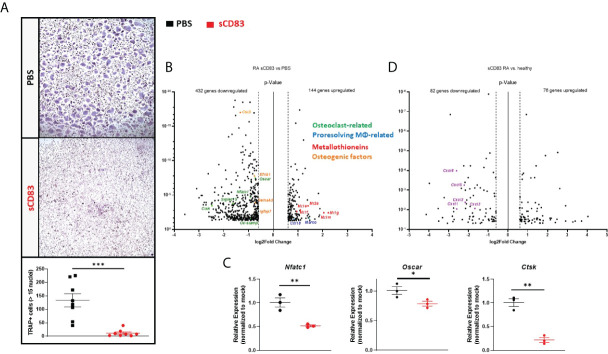
sCD83 potently suppressed the pro-osteoclastogenic phenotype of CD14^+^ cells derived from rheumatoid arthritis (RA) patients. **(A)** Representative images (**A**; upper side) used for the quantification of large multinucleated TRAP^+^ cells with more than 15 nuclei (**A**; lower side) with *n* = 8. **(B)** Volcano plot of RNA sequencing analyses of sCD83- *vs*. phosphate-buffered saline(PBS)-treated osteoclast cultures generated from CD14^+^ cells of RA patients on day 7 from three independent samples per condition. The dots depicted on the right-hand side of the log_2_fold change 0 value represent significantly upregulated transcripts, while the dots on the left-hand side represent significantly downregulated transcripts with log_2_FC ≥0.6, respectively. Specific dot colors classify the transcripts into characteristic groups: green = osteoclast-related, blue = transcripts associated with alternatively activated M2-like MΦ, red = metallothioneins, and orange = transcripts which are associated with osteoblast formation. **(C)** Verification of the RNA sequencing data by RT-PCR analyses regarding the expression of osteoclast-related transcripts from one representative experiment (*n* = 3). **(D)** Volcano plot of RNA sequencing analysis of sCD83-treated osteoclast cultures derived from RA patients compared to sCD83-treated cultures from healthy donors on day 7 from three independent samples per condition. The dots depicted on the right-hand side of the log_2_FC 0 value represent significantly upregulated genes, while the dots on the left-hand side represent significantly downregulated genes with log_2_FC ≥0.6, respectively. The purple dots highlight transcripts of the CXCL-family. **(A)** Mann–Whitney test and **(C)** Student’s *t*-test.

Next, we wanted to find out if sCD83 also modulates osteoclastogenesis in RA patients. sCD83 (25 µg/ml) or PBS was supplemented on a daily base during osteoclast differentiation again, and analogous experiments were performed as described above for healthy donor samples. As depicted in **Figure 4A**, the presence of sCD83 strikingly reduced the number of large multinucleated osteoclasts. The samples were used for subsequent RNA sequencing analyses again. Reportedly, RA patient-derived monocytes are characterized by a pro-inflammatory and pro-osteoclastogenic phenotype (40, 41), and indeed the RNA sequencing analyses of sCD83- or mock-treated osteoclasts generated from RA patients revealed an altered transcriptome (**Figure 4B**) when compared to the analyses of cells derived from healthy donors (**Figure 3B**). A significant downregulation of 432 transcripts, concomitantly with a significant upregulation of 144 gene transcripts, was observed in sCD83 samples (**Figure 4B**). To start with, the suppression of osteoclast-related transcripts, such as *Oscar*, *Nfatc1*, *Siglec15*, *Ctsk*, and *Dc-Stamp* by sCD83 was very prominent and consistent with the analyses of healthy donor-derived cells. This was also the case for osteogenic transcripts, such as *Cst3*, *Efnb1*, *Sema4d*, and *Igfbp7* (**Figure 4B**). These sequencing data were verified by RT-PCR analyses again, as shown for *Nfatc1*, *Oscar*, *Mmp9*, and *Ctsk*. All of these transcripts were significantly reduced in sCD83-treated cultures (**Figure 4C**). It is noteworthy that the metallothionein-specific transcripts were also upregulated by sCD83 in RA patient-derived cells, further highlighting their role in sCD83-induced regulatory mechanisms during osteoclast formation (**Figure 4B**). In sharp contrast, specific alternatively activated M2-like MΦ-related markers, which were prominently upregulated by sCD83 in osteoclast cultures derived from healthy donors, were no longer upregulated in RA patient-derived samples (*i*.*e*., *Mrc1*, *Cd36*, *Mertk*, *Mrc2*, *Msr1*, and *Il-10*).

Although the anti-osteoclastogenic effect of sCD83 was very prominent in RA patient-derived samples, our sequencing data indicate that specific transcripts may be differentially regulated when comparing among healthy individuals and RA patients. In order to investigate this hypothesis in detail, the RNA sequencing analyses of sCD83-treated cells from healthy donors and those ones derived from RA patients were assessed against each other. **Figure 4D** shows that 82 gene transcripts were significantly downregulated, while 76 transcripts were significantly upregulated in sCD83-treated RA patient-derived samples when compared to sCD83-treated healthy donor-derived osteoclasts (**Figure 4D**). Interestingly, we have identified a broad downregulation of RNAs related to the CXCL protein family, including *Cxcl1*, *Cxcl2*, *Cxcl3*, *Cxcl5* and *Cxcl6* in RA-derived samples after sCD83 treatment, which was not the case in healthy donor-derived samples (**Figure 4D**).

In summary, our data described above support the conclusion that sCD83 has striking anti-osteoclastogenic properties not only in cells derived from healthy donors but also in cells derived from RA patients.

### The TRIF pathway is crucial for the sCD83-induced immune regulatory mechanisms downstream to TLR4

**Figure 5:**
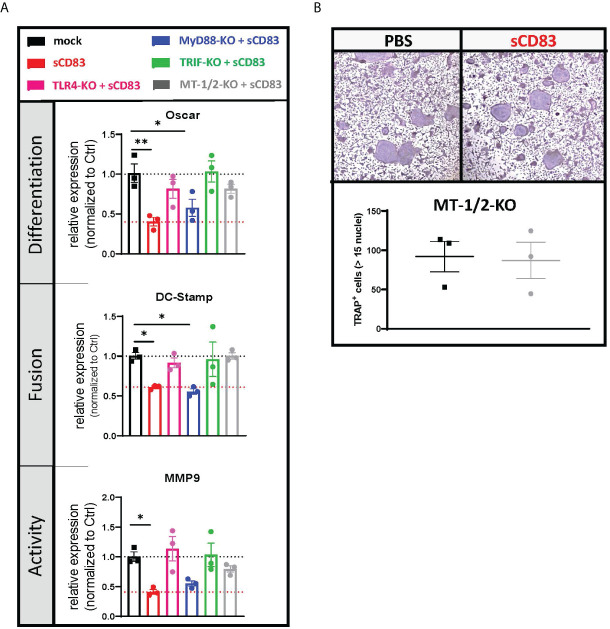
Downstream of TLR4, sCD83 acts *via* the TRIF-mediated signaling cascade in a MyD88-independent manner. **(A)** The mRNA levels of *Oscar*, *Dc-stamp*, and *Mmp9* were analyzed *via* RT-PCR, and Rpl4 was used as a housekeeping gene. Relative expression of sCD83-treated cultures was normalized to the corresponding phosphate-buffered controls (PBS) (with *n* = 3 each). **(B)** Representative images (upper side) used for the quantification of large multinucleated TRAP^+^ cells with more than 15 nuclei (lower side) generated from nucleofected cells, where MT-1 and MT-2 were knocked down by CRISPR/Cas9 with *n* = 3, respectively. Panel **(A)** was tested for significance using ordinary one-way ANOVA against the control condition (black) and panel **(B)** by Student’s *t*-test.

While the binding of LPS to the TLR complex stimulates a pro-inflammatory immune response, sCD83 induces an anti-inflammatory cascade leading to the induction of IDO and IL-10 (42). However, the sCD83-induced signaling downstream of TLR4 is unknown. To unravel these signaling events, we knocked down crucial molecules downstream of the TLR4 signaling cascade including TLR4 itself, MyD88, and TRIF (Ticam1) in murine BMDCs using the CRISPR/Cas9-technique. Subsequently, these precursor cells were used for osteoclastogenesis experiments in the presence of sCD83 (25 µg/ml) or the corresponding PBS control. In addition, we have also deleted the murine metallothioneins MT-1 and MT-2 since we learned from our transcriptome analyses that MTs are involved in sCD83-mediated regulatory circuits (see **Figures 3B**, **4B**). The CRISPR/Cas9-mediated knockout efficiency was determined by the T7 surveyor assay and revealed an efficacy of 40% for TRIF, 85% for MyD88, 99% for TLR4, and 70/55% for MT-1/MT-2, respectively (**Supplementary Figure S2**). **Figure 5A** shows the effects of specific knockdowns with respect to the expression of osteoclast-related transcripts in the presence or absence of sCD83. In control cells, the representative transcripts for differentiation (Oscar), fusion (DC-Stamp), and activity (MMP9) are expressed on a regular level and used as standard control (see **Figure 5A**, black bars). As expected, when sCD83 was added to these control cells, all transcripts were downregulated (see **Figure 5A**, red bars). If TLR4 was deleted, sCD83 had no effect anymore, clearly showing that TLR4 is crucial for the regulatory function of sCD83 (see **Figure 5A**, magenta bars). When we knocked down MyD88, sCD83 was still able to induce its anti-osteoclastogenic effects, indicating that MyD88 is not crucially involved (see **Figure 5A**, blue bars). Finally, when TRIF was deleted, the sCD83-mediated effects were completely abrogated, indicating that TRIF is absolutely essential downstream of the TLR4 receptor (see **Figure 5A**, green bars). TRIF induces the production of type I interferons, which leads to the induction of IDO and, consequently, to the expansion of Tregs. As shown in **Supplementary Figure S3**, sCD83 indeed induces *IFNβ* production over time in human-derived cells, further supporting the role of TRIF in sCD83-mediated signaling events and finally leading to the expansion of Tregs as we and others have shown (43).

### Role of metallothioneins MT-1 and MT-2 in sCD83-induced suppression of osteoclasts

As the expression analyses indicated that metallothioneins are regulated by sCD83, we knocked down metallothioneins MT-1 and MT-2 in murine BMDCs and tested whether the sCD83-induced effects in osteoclasts were affected (**Figure 5A**). On day 4, the number of large multinucleated osteoclasts was determined by TRAP staining. As depicted in the **Figure 5B**, the sCD83-mediated anti-osteoclastogenic effects were completely abrogated when metallothioneins were knocked down. Comparable results were obtained when TLR4 or TRIF knockdown cells were used (**Figure 5A**). Thus, these results clearly show that the TLR4–TRIF pathway, in combination with metallothioneins, is mechanistically important for the inhibition of osteoclasts by sCD83.

## Discussion

In the first part of the project, we investigated the therapeutic potential of sCD83 in established arthritis. Previous studies have shown that the cell-specific knockout of CD83 induces strong pro-inflammatory immune responses (11, 13, 14). Conversely, we observed an accelerated resolution of established arthritis already after short-term treatment with sCD83. According to the histological analyses, the major impact of sCD83 treatment was the clearance of inflammatory cells from the joints. We hypothesize two scenarios for these fast effects of sCD83: The first one is related to induction of the enzyme IDO, which inhibits effector T cells (17). In accordance, *Foxp3* mRNA expression was upregulated in the joints of sCD83-treated mice, indicating the accumulation of Tregs. The second one is related to the striking upregulation of alternatively activated macrophage transcripts observed in our RNA sequencing analyses, suggesting a phenotypic switch towards regulatory macrophages that fosters the resolution of inflammation (44). In support of this concept, mRNA expression of macrophage-derived pro-inflammatory cytokines was decreased in the synovial tissue of sCD83-treated mice.

Apart from the pro-resolving effects of sCD83 on inflammation, our data showed that the molecule is a potent suppressor for osteoclasts. One dose of sCD83 was already sufficient to reduce the number of osteoclasts and downregulate the osteoclast-related genes. Since osteoclasts express TLR4 on their surface and sCD83 has been shown to bind to the TLR4, these cells can directly respond to sCD83 (45). Conversely, the knockout of CD83 from monocyte-lineage cells significantly increased the number of osteoclasts and osteoclast-related transcripts, such as *DC-Stamp* and *OC-Stamp*, indicating increased osteoclast fusion capacities. In this respect, Yagi et al. identified DC-Stamp as a pivotal player during osteoclast fusion, wherein the depletion of DC-Stamp resulted in the complete abrogation of osteoclast fusion and resorptive activity (46). Analogously, OC-Stamp also plays a crucial role for osteoclast fusion. Therefore, their upregulation, as observed in the current work, correlated with the increased number of large multinucleated TRAP^+^ cells (47). Similar effects were seen in human osteoclasts, both from healthy controls and from RA patients. CD83 knockdown led to significantly enhanced formation of osteoclasts, along with significantly increased levels of *Trap*, *Oscar*, and *Mmp9*. Whereas upregulation of TRAP and MMP9 is associated with increased resorption activity (48), OSCAR represents a costimulatory receptor molecule on osteoclast precursor cells (49). These findings were somehow surprising since mCD83 was not known to play an important role during osteoclastogenesis. However, as shown here, when mCD83 is missing, precursor cells acquire an osteoclastogenic phenotype not only in cells derived from healthy donors but also from those derived from a RA patient.

RNA sequencing analyses of osteoclasts showed the downregulation of osteoclast-related transcripts such as *Nfatc1*, *Ctsk*, *Mmp9*, *OcStamp*, and *Trap* by sCD83, while markers associated with alternative differentiation of macrophages were induced. In addition, *Siglec 15* was blocked by sCD83, a molecule that has recently been shown to play a role in osteoclast formation (29).

The upregulation of alternatively activated macrophage transcripts was surprising since the supplementation with RANKL in our cultures rather inhibits such anti-osteoclastogenic differentiation processes (50). Skewing of macrophage differentiation by sCD83 is, for instance, reflected by increased RNA levels for *Klf4* (51) and the increased *Il-10* expression. Of particular note is that Stat6 was also upregulated in the sCD83-treated osteoclast cultures. As reported by Moreno and colleagues, Stat6 activity induces an irreversible inhibition of the RANKL-induced osteoclast differentiation process (52). Therefore, we assume that, up from the first supplementation with sCD83, macrophages are driven into alternatively activated macrophages with anti-osteoclastogenic and anti-inflammatory capacities (53).

We also observed a significant upregulation of metallothioneins by sCD83. Metallothioneins have regulatory effects in arthritis by the induction of pro-resolving mediators, such as TGFβ and a switch of the Th17/Treg balance (54, 55). Mechanistically, metallothioneines interfere with the free Zn^2+^ ions (30, 31). Zn^2+^ accumulates within the cytoplasm upon TLR4 stimulation and facilitates the induction of pro-inflammatory cytokine expression (30). The binding of Zn^2+^ by metallothioneines neutralizes these pro-inflammatory effects and stabilizes IRF3 translocation into the nucleus and the induction of IFNβ, which, in turn, promotes anti-inflammatory signals, *e*.*g*., *via* the induction of IDO and TGFβ (17, 56). This is an interesting mechanistic link to sCD83 since we and others have previously shown that IDO as well as TGFβ are crucial mediators for the observed sCD83-mediated anti-inflammatory effects *in vitro* as well as *in vivo* (8, 57). Moreover, knockdown of metallothioneines MT-1 and MT-2 completely abolished the anti-osteoclastogenic effect of sCD83. Our effects of sCD83 on the transcriptional profile of monocytes from healthy individuals could be reproduced in cells derived from RA patients. Thus, osteoclast-related transcripts were significantly downregulated, while metallothioneines were significantly upregulated upon sCD83 treatment also in RA patients. When comparing sCD83-induced gene expression profiles in cells derived from healthy controls and RA patients, we observed a prominent downregulation of the CXCL family members in RA patients, but not in samples from healthy donors. In this context, it has been reported that CXCLs strongly promote a pro-osteoclastogenic environment, while their depletion negatively affects osteoclastogenesis (58).

The anti-inflammatory effects of sCD83 are based on binding to the TLR4/MD2 complex (42). Thus, we aimed to investigate the signaling cascade downstream of TLR4 in more detail. The deletion of TLR4 completely abrogated the anti-osteoclastogenic effects of sCD83. Of note is that a very same outcome was observed when sCD83 was applied to osteoclast cultures in the presence of the TLR4-inhibitor CLI-095 (data not shown). Downstream of TLR4, the knockdown of MyD88, which is the adaptor molecule of the pro-inflammatory LPS-induced TLR4-signaling cascade (59), did not affect the inhibitory effect of sCD83. Of note is that we obtained comparable results with cells derived from MyD88^-/-^ mice (data not shown). In contrast, the knockdown of TRIF resulted in the complete abrogation of the anti-osteoclastogenic effects of sCD83. TRIF is associated with anti-inflammatory and tolerogenic mechanisms, *e*.*g*., *via* the induction of type I interferons, which, in turn, induce TGFβ and IDO expression (60). This finding extends by far our previous work, where we have only shown that sCD83 induces IDO as well as TGFβ, and the inhibition of both abrogates sCD83-induced regulatory mechanism *in vitro* as well as *in vivo* (8, 11).

**Figure 6:**
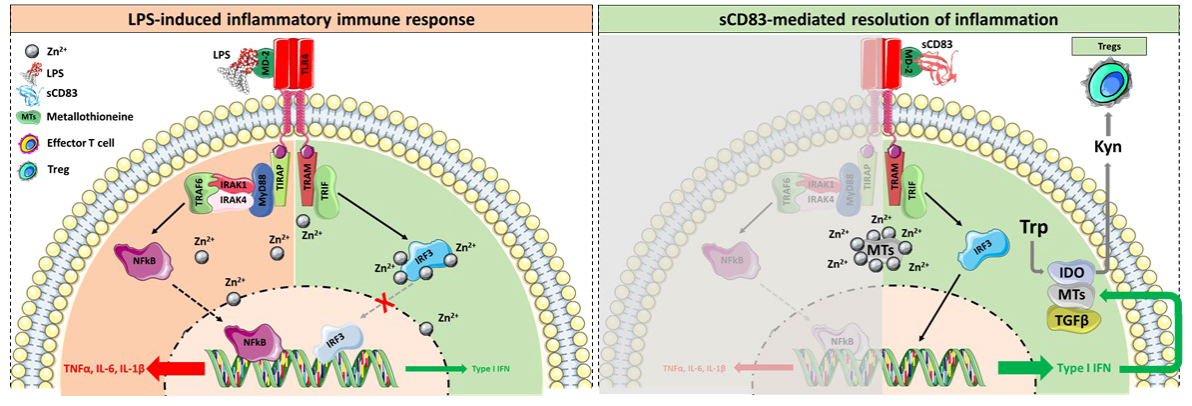
Graphical summary of the sCD83-induced regulatory mechanism. (Upper side) Binding of LPS to the TLR4/MD-2 complex results in the activation of the MyD88 signaling cascade, the translocation of NFκB into the nucleus, and the subsequent expression of pro-inflammatory cytokines. Concomitantly, TLR4 stimulation leads to activation-induced Zn^2+^ influx into the cytosol, a crucial event for the dissociation of NFκB from its inhibitor, thus facilitating its translocation into the nucleus. Moreover, Zn^2+^ potently inhibited the TRIF-mediated anti-inflammatory signaling pathway by blocking the IRF3 activity. (Lower side) In sharp contrast, the binding of sCD83 to the TLR4/MD-2 complex fosters the resolution of inflammation in a TRIF-dependent manner. In addition, sCD83 strongly induces the expression of MTs, which bind free Zn^2+^, thereby promoting the translocation of IRF3 into the nucleus and the subsequent induction of type I IFN. Type I IFNs are potent inducers of IDO and TGF, both shown to be crucial for the sCD83-induced differentiation of Tregs and the long term-resolution of inflammation. Herein IDO converts the amino acid tryptophan (Trp), which is indispensable for effector T cell proliferation and survival, into Kynurenine (Kyn), a potent inducer of Tregs *via* the Ahr pathway. In addition, type I IFNs further boost the expression of MTs and thus stabilize the TRIF-associated resolving pathway over the pro-inflammatory MyD88 cascade.

In summary, we show that (i) sCD83 is not only protective using a prophylactic setting (8) but also applying a systemic administration route, (ii) we have successfully translated our murine data into the human system using osteoclast precursor cells from healthy donors and (iii) from RA patients, which is an important finding in respect to the future clinical applicability and efficacy, (iv) sCD83 is a potent modulator of macrophages towards a pro-resolving and anti-arthritic phenotype, which is very important for the resolution of inflammation, and (v) mechanistically, sCD83 mediates its anti-inflammatory and anti-osteoclastogenic effects *via* the TRIF signaling pathway and in conjunction with metallothioneines. A graphical summary comparing LPS- *versus* sCD83-induced signaling events is shown in **Figure 6**. In sharp contrast to the LPS-induced pro-inflammatory signaling cascade *via* the MyD88 pathway (**Figure 6**, upper graph), sCD83 effects are mediated *via* the TRIF pathway (**Figure 6**, lower graph). In addition, under inflammatory conditions, the intracellular concentrations of free Zn^2+^ are upregulated upon TLR4 stimulation in order to facilitate NF-kB activation and the expression of proinflammatory cytokines, such as IL-6, TNFα, and IL-1β. Concomitantly, Zn^2+^ represents a negative regulator of the TRIF/IRF3 pathway, thereby further enhancing the inflammatory immune response (31). However, metallothioneines possess strong Zn^2+^-binding capacities, thereby limiting its intracellular availability and activity and thus stabilizing the TRIF pathway along with a downstream induction of type I IFNs (61). Type I IFNs, in turn, induce the enzyme IDO, which inhibits effector T cell activity, induces regulatory T cells, and blocks osteoclast formation (60, 62). Thus, these data show that CD83 induces a pro-resolving and anti-osteoclastogenic environment leading to the inhibition of osteoclast-mediated bone resorption.

## Data availability statement

The data presented in the study are deposited in the GEO repository, accession number GSE207872 (https://www.ncbi.nlm.nih.gov/geo/query/acc.cgi?acc=GSE207872).

## Ethics statement

The studies involving human participants were reviewed and approved by Ethik-Komission der FAU: 30_20B and 431_19B. The patients/participants provided their written informed consent to participate in this study. The animal study was reviewed and approved by the Goverment of Unterfranken, Würzburg DMS-2532-2-191. Written informed consent was obtained from the owners for the participation of their animals in this study.

## Author contributions

DR and AS designed the experiments, evaluated the results, and wrote the manuscript. DR, LS, KP-M, SA, DA, AW, EZ, PM-Z, LN, and EJ acquired data and helped with the experiments. MF and MK supervised and performed bioinformatical and statistical analyses of RNAseq data and data curation. DR, DA, AS, AB, KP-M, LS, SA, SF, GS, AW, EZ, MF, MK, LN, and TB interpreted data and revised the manuscript. AS and DR acquired funding. All authors contributed to the article and approved the submitted version.

## Funding

This work was funded and supported by the Deutsche Forschungsgemeinschaft (DFG) grant SFB1181 via projects B03 (to AS), A01 (to AB and GS), and Z02 (to TB), the Interdisciplinary Center for Clinical Research (IZKF) at the University Hospital of the University of Erlangen-Nuremberg (ELAN P077), German Federal Ministry of Education and Research (BMBF) CompLS program grant 031L0262C (to MK), and Fraunhofer Cluster of Excellence Immune-Mediated Diseases (CIMD; to MF and MK). KP-M was funded by the Bavarian Equal Opportunities Sponsorship—Realization Equal Opportunities for Women in Research and Teaching.

## Acknowledgments

We would like to thank Barbara Happich for the preparation of histological samples.

## Conflict of interest

The authors declare that the research was conducted in the absence of any commercial or financial relationships that could be construed as a potential conflict of interest.

## Publisher’s note

All claims expressed in this article are solely those of the authors and do not necessarily represent those of their affiliated organizations, or those of the publisher, the editors and the reviewers. Any product that may be evaluated in this article, or claim that may be made by its manufacturer, is not guaranteed or endorsed by the publisher.

## References

[1] Guo Q Wang Y Xu D Nossent J Pavlos NJ Xu J. Rheumatoid arthritis: pathological mechanisms and modern pharmacologic therapies. Bone Res (2018) 6:15. doi: 10.1038/s41413-018-0016-9 29736302 PMC5920070

[2] McInnes IB Schett G. The pathogenesis of rheumatoid arthritis. N Engl J Med (2011) 365(23):2205–19. doi: 10.1056/NEJMra1004965 22150039

[3] Voigt LF Koepsell TD Nelson JL Dugowson CE Daling JR. Smoking, obesity, alcohol consumption, and the risk of rheumatoid arthritis. Epidemiology (1994) 5(5):525–32. 7986867

[4] Papadaki M Rinotas V Violitzi F Thireou T Panayotou G Samiotaki M. New insights for RANKL as a proinflammatory modulator in modeled inflammatory arthritis. Front Immunol (2019) 10:97. doi: 10.3389/fimmu.2019.00097 30804932 PMC6370657

[5] Bullock J Rizvi SAA Saleh AM Ahmed SS Do DP Ansari RA. Rheumatoid arthritis: A brief overview of the treatment. Med Princ Pract (2018) 27(6):501–7. doi: 10.1159/000493390 PMC6422329 30173215

[6] George MD Baker JF Winthrop K Alemao E Chen L Connolly S. Risk of biologics and glucocorticoids in patients with rheumatoid arthritis undergoing arthroplasty: A cohort study. Ann Intern Med (2019) 170(12):825–36. doi: 10.7326/M18-2217 PMC7197029 31108503

[7] Eckhardt J Kreiser S Dobbeler M Nicolette C DeBenedette MA Tcherepanova IY. Soluble CD83 ameliorates experimental colitis in mice. Mucosal Immunol (2014) 7(4):1006–18. doi: 10.1038/mi.2013.119 24424524

[8] Royzman D Andreev D Stich L Rauh M Bauerle T Ellmann S. Soluble CD83 triggers resolution of arthritis and sustained inflammation control in IDO dependent manner. Front Immunol (2019) 10:633. doi: 10.3389/fimmu.2019.00633 31001257 PMC6455294

[9] Starke C Steinkasserer A Voll RE Zinser E. Soluble human CD83 ameliorates lupus in NZB/W F1 mice. Immunobiology (2013) 218(11):1411–5. doi: 10.1016/j.imbio.2013.06.002 23886695

[10] Zinser E Lechmann M Golka A Lutz MB and Steinkasserer A.. Prevention and treatment of experimental autoimmune encephalomyelitis by soluble CD83. J Exp Med (2004) 200(3):345–51. doi: 10.1084/jem.20030973 PMC2211980 15289503

[11] Grosche L Knippertz I Konig C Royzman D Wild AB Zinser E. The CD83 molecule - an important immune checkpoint. Front Immunol (2020) 11:721. doi: 10.3389/fimmu.2020.00721 32362900 PMC7181454

[12] Krzyzak L Seitz C Urbat A Hutzler S Ostalecki C Glasner J. CD83 modulates b cell activation and germinal center responses. J Immunol (2016) 196(9):3581–94. doi: 10.4049/jimmunol.1502163 26983787

[13] Doebbeler M Koenig C Krzyzak L Seitz C Wild A Ulas T. CD83 expression is essential for treg cell differentiation and stability. JCI Insight (2018) 3(11):e99712. doi: 10.1172/jci.insight.99712 PMC6124443 29875316

[14] Wild AB Krzyzak L Peckert K Stich L Kuhnt C Butterhof A. CD83 orchestrates immunity toward self and non-self in dendritic cells. JCI Insight (2019) 4(20):e126246. doi: 10.1172/jci.insight.126246 PMC6824307 31527313

[15] Hock BD O'Donnell JL Taylor K Steinkasserer A McKenzie JL Rothwell AG. Levels of the soluble forms of CD80, CD86, and CD83 are elevated in the synovial fluid of rheumatoid arthritis patients. Tissue Antigens (2006) 67(1):57–60. doi: 10.1111/j.1399-0039.2005.00524.x 16451202

[16] Lob S Konigsrainer A Schafer R Rammensee HG Opelz G Terness P. Levo- but not dextro-1-methyl tryptophan abrogates the IDO activity of human dendritic cells. Blood (2008) 111(4):2152–4. doi: 10.1182/blood-2007-10-116111 18045970

[17] Chen W. IDO: more than an enzyme. Nat Immunol (2011) 12(9):809–11. doi: 10.1038/ni.2088 21852775

[18] Zaiss MM Axmann R Zwerina J Polzer K Guckel E Skapenko A. Treg cells suppress osteoclast formation: a new link between the immune system and bone. Arthritis Rheum (2007) 56(12):4104–12. doi: 10.1002/art.23138 18050211

[19] Pfeiffer IA Zinser E Strasser E Stein MF Dorrie J Schaft N. Leukoreduction system chambers are an efficient, valid, and economic source of functional monocyte-derived dendritic cells and lymphocytes. Immunobiology (2013) 218(11):1392–401. doi: 10.1016/j.imbio.2013.07.005 23932569

[20] Bouffi C Bony C Courties G Jorgensen C Noel D. IL-6-dependent PGE2 secretion by mesenchymal stem cells inhibits local inflammation in experimental arthritis. PloS One (2010) 5(12):e14247. doi: 10.1371/journal.pone.0014247 21151872 PMC2998425

[21] Dominici M Le Blanc K Mueller I Slaper-Cortenbach I Marini F Krause D. Minimal criteria for defining multipotent mesenchymal stromal cells. the international society for cellular therapy position statement. Cytotherapy (2006) 8(4):315–7. doi: 10.1080/14653240600855905 16923606

[22] Hiatt J Cavero DA McGregor MJ Zheng W Budzik JM Roth TL. Efficient generation of isogenic primary human myeloid cells using CRISPR-Cas9 ribonucleoproteins. Cell Rep (2021) 35(6):109105. doi: 10.1016/j.celrep.2021.109105 33979618 PMC8188731

[23] Freund EC Lock JY Oh J Maculins T Delamarre L Bohlen CJ. Efficient gene knockout in primary human and murine myeloid cells by non-viral delivery of CRISPR-Cas9. J Exp Med (2020) 217(7). doi: 10.1084/jem.20191692 PMC7336301 32357367

[24] Liao Y Smyth GK Shi W. The r package rsubread is easier, faster, cheaper and better for alignment and quantification of RNA sequencing reads. Nucleic Acids Res (2019) 47(8):e47. doi: 10.1093/nar/gkz114 30783653 PMC6486549

[25] Fuchs M Kreutzer FP Kapsner LA Mitzka S Just A Perbellini F. Integrative bioinformatic analyses of global transcriptome data decipher novel molecular insights into cardiac anti-fibrotic therapies. Int J Mol Sci (2020) 21(13):4727. doi: 10.3390/ijms21134727 PMC7370212 32630753

[26] Love MI Huber W Anders S. Moderated estimation of fold change and dispersion for RNA-seq data with DESeq2. Genome Biol (2014) 15(12):550. doi: 10.1186/s13059-014-0550-8 25516281 PMC4302049

[27] Raudvere U Kolberg L Kuzmin I Arak T Adler P Peterson H. g:Profiler: a web server for functional enrichment analysis and conversions of gene lists (2019 update). Nucleic Acids Res (2019) 47(W1):W191–8. doi: 10.1093/nar/gkz369 PMC6602461 31066453

[28] Buchner E Brauer R Schmidt C Emmrich F Kinne RW. Induction of flare-up reactions in rat antigen-induced arthritis. J Autoimmun (1995) 8(1):61–74. doi: 10.1016/S0896-8411(18)30379-2 7734037

[29] Korn MA Schmitt H Angermuller S Chambers D Seeling M Lux UT. Siglec-15 on osteoclasts is crucial for bone erosion in serum-transfer arthritis. J Immunol (2020) 205(10):2595–605. doi: 10.4049/jimmunol.2000472 33020147

[30] Brieger A Rink L Haase H. Differential regulation of TLR-dependent MyD88 and TRIF signaling pathways by free zinc ions. J Immunol (2013) 191(4):1808–17. doi: 10.4049/jimmunol.1301261 23863901

[31] Kimura T Kambe T. The functions of metallothionein and ZIP and ZnT transporters: An overview and perspective. Int J Mol Sci (2016) 17(3):336. doi: 10.3390/ijms17030336 26959009 PMC4813198

[32] Ka MB Daumas A Textoris J Mege JL.. Phenotypic diversity and emerging new tools to study macrophage activation in bacterial infectious diseases. Front Immunol (2014) 5:500. doi: 10.3389/fimmu.2014.00500 25346736 PMC4193331

[33] Soldano S Pizzorni C Paolino S Trombetta AC Montagna P Brizzolara R. Alternatively activated (M2) macrophage phenotype is inducible by endothelin-1 in cultured human macrophages. PloS One (2016) 11(11):e0166433. doi: 10.1371/journal.pone.0166433 27846260 PMC5112853

[34] Zizzo G Hilliard BA Monestier M Cohen PL.. Efficient clearance of early apoptotic cells by human macrophages requires M2c polarization and MerTK induction. J Immunol (2012) 189(7):3508–20. doi: 10.4049/jimmunol.1200662 PMC3465703 22942426

[35] Abu-Amer Y. IL-4 abrogates osteoclastogenesis through STAT6-dependent inhibition of NF-kappaB. J Clin Invest (2001) 107(11):1375–85. doi: 10.1172/JCI10530 PMC209314 11390419

[36] Kapoor N Niu J Saad Y Kumar S Sirakova T Becerra E. Transcription factors STAT6 and KLF4 implement macrophage polarization via the dual catalytic powers of MCPIP. J Immunol (2015) 194(12):6011–23. doi: 10.4049/jimmunol.1402797 PMC4458412 25934862

[37] Han Y You X Xing W Zhang Z Zou W. Paracrine and endocrine actions of bone-the functions of secretory proteins from osteoblasts, osteocytes, and osteoclasts. Bone Res (2018) 6:16. doi: 10.1038/s41413-018-0019-6 29844945 PMC5967329

[38] Weivoda MM Chew CK Monroe DG Farr JN Atkinson EJ Geske JR. Identification of osteoclast-osteoblast coupling factors in humans reveals links between bone and energy metabolism. Nat Commun (2020) 11(1):87. doi: 10.1038/s41467-019-14003-6 31911667 PMC6946812

[39] Ye C Hou W Chen M Lu J Chen E Tang L. IGFBP7 acts as a negative regulator of RANKL-induced osteoclastogenesis and oestrogen deficiency-induced bone loss. Cell Prolif (2020) 53(2):e12752. doi: 10.1111/cpr.12752 31889368 PMC7046308

[40] McGarry T Hanlon MM Marzaioli V Cunningham CC Krishna V Murray K. Rheumatoid arthritis CD14(+) monocytes display metabolic and inflammatory dysfunction, a phenotype that precedes clinical manifestation of disease. Clin Transl Immunol (2021) 10(1):e1237. doi: 10.1002/cti2.1237 PMC7815439 33510894

[41] Shang W Zhao LJ Dong XL Zhao ZM Li J Zhang BB. Curcumin inhibits osteoclastogenic potential in PBMCs from rheumatoid arthritis patients via the suppression of MAPK/RANK/c-Fos/NFATc1 signaling pathways. Mol Med Rep (2016) 14(4):3620–6. doi: 10.3892/mmr.2016.5674 PMC5042742 27572279

[42] Horvatinovich JM Grogan EW Norris M Steinkasserer A Lemos H Mellor AL. Soluble CD83 inhibits T cell activation by binding to the TLR4/MD-2 complex on CD14(+) monocytes. J Immunol (2017) 198(6):2286–301. doi: 10.4049/jimmunol.1600802 PMC5337811 28193829

[43] De Luca A Montagnoli C Zelante T Bonifazi P Bozza S Moretti S. Functional yet balanced reactivity to candida albicans requires TRIF, MyD88, and IDO-dependent inhibition of rorc. J Immunol (2007) 179(9):5999–6008. doi: 10.4049/jimmunol.179.9.5999 17947673

[44] Kim H Wang SY Kwak G Yang Y Kwon IC Kim SH. Exosome-guided phenotypic switch of M1 to M2 macrophages for cutaneous wound healing. Adv Sci (Weinh) (2019) 6(20):1900513. doi: 10.1002/advs.201900513 31637157 PMC6794619

[45] Yim M. The role of toll-like receptors in osteoclastogenesis. J Bone Metab (2020) 27(4):227–35. doi: 10.11005/jbm.2020.27.4.227 PMC7746484 33317226

[46] Yagi M Miyamoto T Sawatani Y Iwamoto K Hosogane N Fujita N. DC-STAMP is essential for cell-cell fusion in osteoclasts and foreign body giant cells. J Exp Med (2005) 202(3):345–51. doi: 10.1084/jem.20050645 PMC2213087 16061724

[47] Yang M Birnbaum MJ MacKay CA Mason-Savas A Thompson B Odgren PR. Osteoclast stimulatory transmembrane protein (OC-STAMP), a novel protein induced by RANKL that promotes osteoclast differentiation. J Cell Physiol (2008) 215(2):497–505. doi: 10.1002/jcp.21331 18064667 PMC2762860

[48] Franco GC Kajiya M Nakanishi T Ohta K Rosalen PL Groppo FC. Inhibition of matrix metalloproteinase-9 activity by doxycycline ameliorates RANK ligand-induced osteoclast differentiation in vitro and in vivo. Exp Cell Res (2011) 317(10):1454–64. doi: 10.1016/j.yexcr.2011.03.014 PMC3115670 21420951

[49] Nemeth K Schoppet M Al-Fakhri N Helas S Jessberger R Hofbauer LC. The role of osteoclast-associated receptor in osteoimmunology. J Immunol (2011) 186(1):13–8. doi: 10.4049/jimmunol.1002483 21172874

[50] Huang R Wang X Zhou Y Xiao Y. RANKL-induced M1 macrophages are involved in bone formation. Bone Res (2017) 5:17019. doi: 10.1038/boneres.2017.19 29263936 PMC5645773

[51] Liao X Sharma N Kapadia F Zhou G Lu Y Hong H. Kruppel-like factor 4 regulates macrophage polarization. J Clin Invest (2011) 121(7):2736–49. doi: 10.1172/JCI45444 PMC3223832 21670502

[52] Moreno JL Kaczmarek M Keegan AD Tondravi M. IL-4 suppresses osteoclast development and mature osteoclast function by a STAT6-dependent mechanism: irreversible inhibition of the differentiation program activated by RANKL. Blood (2003) 102(3):1078–86. doi: 10.1182/blood-2002-11-3437 12689929

[53] Yao Y Cai X Ren F Ye Y Wang F Zheng C. The macrophage-osteoclast axis in osteoimmunity and osteo-related diseases. Front Immunol (2021) 12:664871. doi: 10.3389/fimmu.2021.664871 33868316 PMC8044404

[54] Sun J Li L Li L Ding L Liu X Chen X,. Metallothionein-1 suppresses rheumatoid arthritis pathogenesis by shifting the Th17/Treg balance. Eur J Immunol (2018) 48(9):1550–62. doi: 10.1002/eji.201747151 30055006

[55] Youn J Hwang SH Ryoo ZY Lynes MA Paik DJ Chung HS. Metallothionein suppresses collagen-induced arthritis via induction of TGF-beta and down-regulation of proinflammatory mediators. Clin Exp Immunol (2002) 129(2):232–9. doi: 10.1046/j.1365-2249.2002.01922.x PMC1906452 12165078

[56] Kumaran Satyanarayanan S El Kebir D Soboh S Butenko S Sekheri M Saadi J,. IFN-beta is a macrophage-derived effector cytokine facilitating the resolution of bacterial inflammation. Nat Commun (2019) 10(1):3471. doi: 10.1038/s41467-019-10903-9 31375662 PMC6677895

[57] Bock F Rossner S Onderka J Lechmann M Pallotta MT Fallarino F. Topical application of soluble CD83 induces IDO-mediated immune modulation, increases Foxp3+ T cells, and prolongs allogeneic corneal graft survival. J Immunol (2013) 191(4):1965–75. doi: 10.4049/jimmunol.1201531 23851696

[58] Hardaway AL Herroon MK Rajagurubandara E Podgorski I.. Marrow adipocyte-derived CXCL1 and CXCL2 contribute to osteolysis in metastatic prostate cancer. Clin Exp Metastasis (2015) 32(4):353–68. doi: 10.1007/s10585-015-9714-5 PMC4393805 25802102

[59] Lin X Kong J Wu Q Yang Y Ji P. Effect of TLR4/MyD88 signaling pathway on expression of IL-1beta and TNF-alpha in synovial fibroblasts from temporomandibular joint exposed to lipopolysaccharide. Mediators Inflammation (2015) 2015:329405. doi: 10.1155/2015/329405 PMC4354974 25810567

[60] Mbongue JC Nicholas DA Torrez TW Kim NS Firek AF Langridge WH. The role of indoleamine 2, 3-dioxygenase in immune suppression and autoimmunity. Vaccines (Basel) (2015) 3(3):703–29. doi: 10.3390/vaccines3030703 PMC4586474 26378585

[61] Sato M Yamaki J Oguro T Yoshida T Nomura N Nakajima K. Metallothionein synthesis induced by interferon alpha/beta in mice of various zinc status. Tohoku J Exp Med (1996) 178(3):241–50. doi: 10.1620/tjem.178.241 8727706

[62] Zaiss MM Frey B Hess A Zwerina J Luther J Nimmerjahn F. Regulatory T cells protect from local and systemic bone destruction in arthritis. J Immunol (2010) 184(12):7238–46. doi: 10.4049/jimmunol.0903841 20483756

