# Supplementary material for: The soluble CD83 protein prevents bone destruction by inhibiting the formation of osteoclasts and inducing resolution of inflammation in arthritis
Source: Front Immunol. 2022 Aug 8;13:936995. doi: 10.3389/fimmu.2022.936995 (PMC9393726; doi:10.3389/fimmu.2022.936995)
Supplement: Supplementary file 1 [file DataSheet_1.pdf]

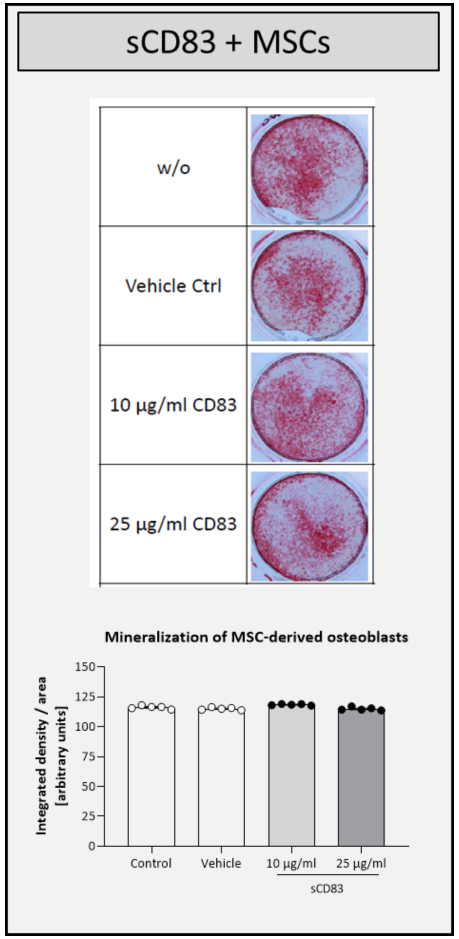

Supplementary Figure 1: Representative slides of osteoblast cultures derived from MSCs in the presence of sCD83 (10 µg/ml and 25 µg/ml), PBS or without any additives (upper side). Mineralization activity expressed as integrated density/area (lower side).

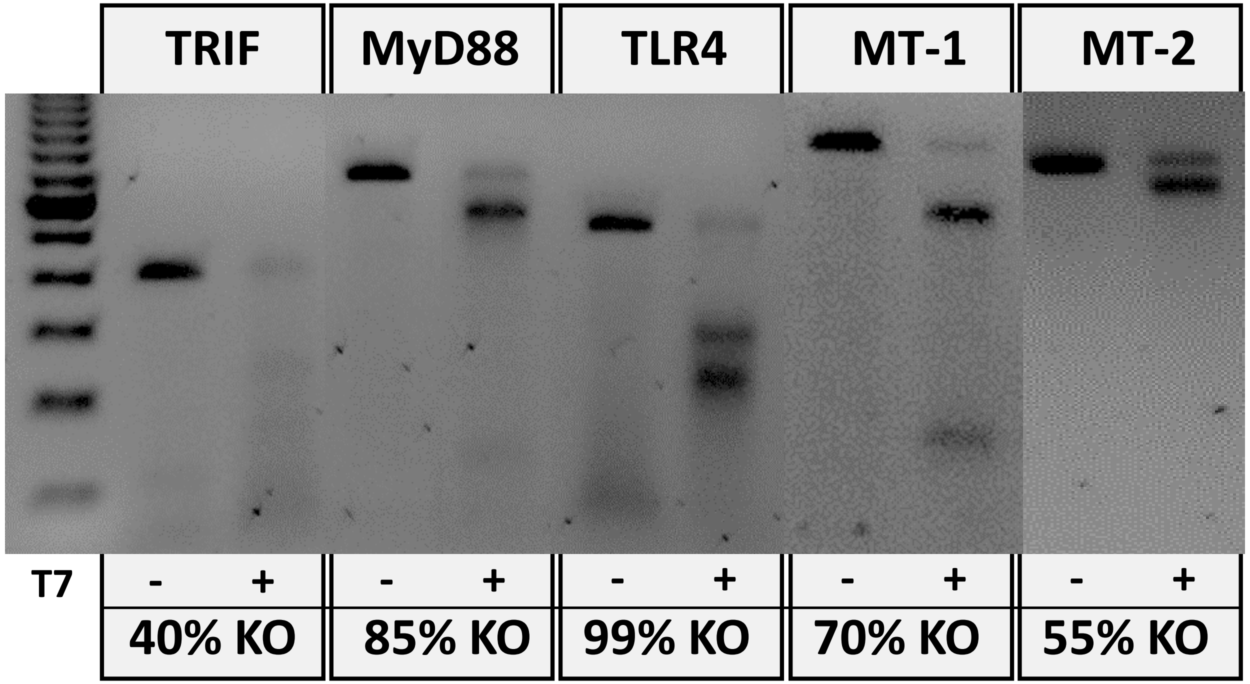

Supplementary Figure 2: Specific gene targeting efficacy for: TICAM1 (TRIF), MyD88, TLR4, MT-1 and MT-2 as determined by T7-Endonuclease-mediated digestion of heteroduplexes.

Supplementary Fig. 3

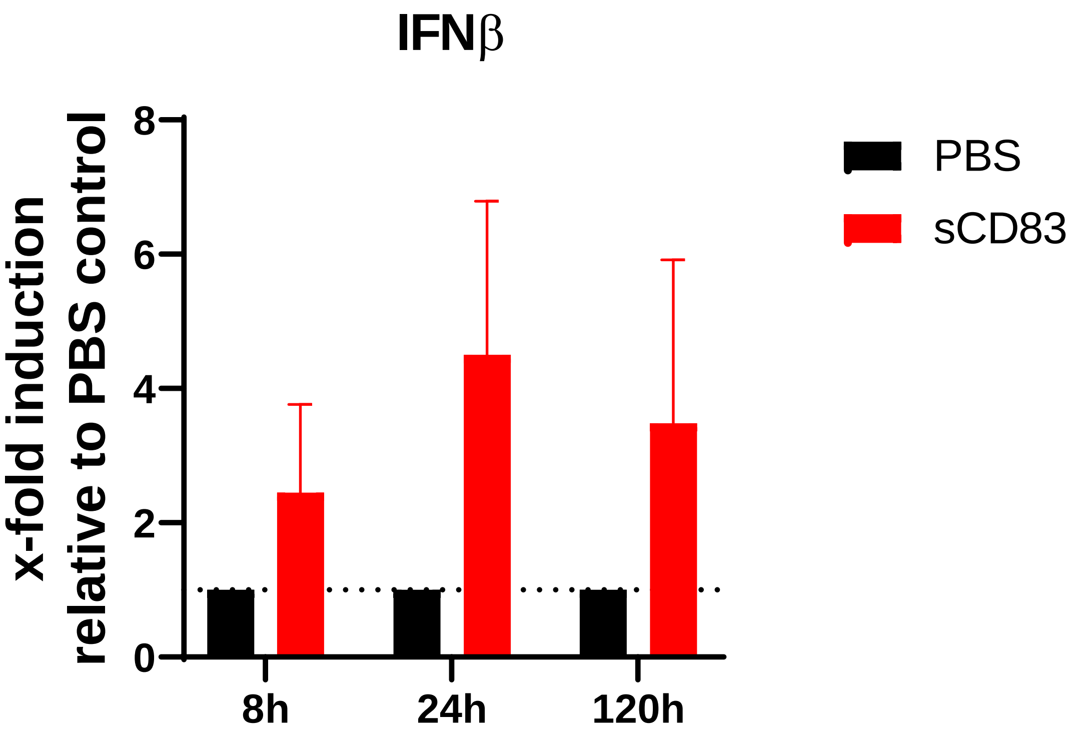

Supplementary Figure 3: RT-PCR analyses of Ifnβ expression in human-derived PBMC-cultures were performed 8h, 24h and 120h after the induction of osteoclastogenesis with n=3. Data are shown as mean ± SEM normalized to the corresponding mock-controls.
